# BioGateway: a semantic systems biology tool for the life sciences

**DOI:** 10.1186/1471-2105-10-S10-S11

**Published:** 2009-10-01

**Authors:** Erick Antezana, Ward Blondé, Mikel Egaña, Alistair Rutherford, Robert Stevens, Bernard De Baets, Vladimir Mironov, Martin Kuiper

**Affiliations:** 1 Dept of Plant Systems Biology, VIB, Gent, Belgium; 2 Dept of Molecular Genetics, Ghent University, Belgium; 3 Dept of Applied Mathematics, Biometrics and Process Control, Ghent University, Belgium; 4 School of Computer Science, University of Manchester, UK; 5 Glasgow, UK; 6 Dept. of Biology, Norwegian University of Science and Technology, Trondheim, Norway

**Keywords:** Resource Description Framework, Transitive Closure, SPARQL Query, Gene Ontology Annotation, Resource Description Framework Graph

## Abstract

**Background:**

Life scientists need help in coping with the plethora of fast growing and scattered knowledge resources. Ideally, this knowledge should be integrated in a form that allows them to pose complex questions that address the properties of biological systems, independently from the origin of the knowledge. Semantic Web technologies prove to be well suited for knowledge integration, knowledge production (hypothesis formulation), knowledge querying and knowledge maintenance.

**Results:**

We implemented a semantically integrated resource named BioGateway, comprising the entire set of the OBO foundry candidate ontologies, the GO annotation files, the SWISS-PROT protein set, the NCBI taxonomy and several in-house ontologies. BioGateway provides a single entry point to query these resources through SPARQL. It constitutes a key component for a Semantic Systems Biology approach to generate new hypotheses concerning systems properties. In the course of developing BioGateway, we faced challenges that are common to other projects that involve large datasets in diverse representations. We present a detailed analysis of the obstacles that had to be overcome in creating BioGateway. We demonstrate the potential of a comprehensive application of Semantic Web technologies to global biomedical data.

**Conclusion:**

The time is ripe for launching a community effort aimed at a wider acceptance and application of Semantic Web technologies in the life sciences. We call for the creation of a forum that strives to implement a truly semantic life science foundation for Semantic Systems Biology.

Access to the system and supplementary information (such as a listing of the data sources in RDF, and sample queries) can be found at http://www.semantic-systems-biology.org/biogateway.

## Background

Systems Biology aims to offer a holistic view of the way in which biological systems work. Systems Biology is an integrative biology, and is already instrumental in rationalising the exploitation of the so-called "-omics" technologies. These technologies are producing massive amounts of biological data which are often stored in disparate, specialised repositories that may not always abide by common standards for data formats [1–3]. This wealth of information is difficult to exploit, since there are complexities involved in combining various data sources to answer even relatively simple questions [4].

A powerful integration of available biological data and knowledge needs an efficient information retrieval and management system. Semantic Web technologies are designed to meet this challenge, and the Semantic Web promises an infrastructure that comprises machine understandable content and therefore a World Wide Web consisting of linked data instead of documents alone. Indeed, computational systems based on a semantic integration of raw data and ontological relationships will provide a sophisticated framework to interrogate and retrieve pertinent information. Integrated knowledge resources may even allow the deployment of advanced computational reasoning approaches [5] in order to generate new hypotheses about the functionality of biological systems.

We are witnessing a growing acceptance of Semantic Web technologies for knowledge integration in the life sciences. This is illustrated by the existence of a W3C special interest group [6] (Semantic Web Health Care and Life Sciences Interest Group – HCLS IG) and many other projects and languages that exploit semantic technologies. For example, the Resource Description Framework (RDF) [7] and the Web Ontology Language (OWL) [8] can be used to represent biological information and the SPARQL query language [9] can be used for the retrieval of information. Semantic Web technologies have the potential to add a new dimension of knowledge integration to Systems Biology that is expected to be among the early adopters of these technologies [10].

To explore the potential for Systems Biology we constructed BioGateway [11], a system built on an RDF store that aggregates bio-ontologies and other biological information sources. Mathematical modelling lies at the core of Systems Biology. By integrating a systems network with a mathematical model, one can simulate the behaviour of the network, and thus predict the outcome of new experiments. Systems Biology is, however, also an integrative approach, and with BioGateway we add a semantic foundation for data integration. Semantic Knowledge Bases (KBs) offer a querying and reasoning component, through which new hypotheses about the system and its components can be obtained. We call this combination *Semantic Systems Biology* (SSB), a form of systems biology in which new hypotheses concerning a biological system are generated through queries and reasoning on integrated data, as opposed to being generated through a mathematical model. We believe that Semantic Systems Biology may provide a powerful complement to the mathematical model-based Systems Biology.

## Results

### BioGateway data model

#### RDF-ing the resources

A suitable data representation is necessary to share and uniformly query the RDF integrated repository. Although different means exist to translate ontologies in the Open Biomedical Ontologies Format (OBOF) to several representations [12], there is no accepted mapping of OBO ontologies to RDF. The mapping used for BioGateway is an extended and improved version of the mapping from the ONTO-PERL suite [13]: it was devised to retain a high-fidelity conversion (*i.e*. no information is lost) and to facilitate querying. The ONTO-PERL suite is a Perl API that can be used to programmatically manipulate ontologies in OBO format. ONTO-PERL offers the possibility of translating an ontology in OBO format to any of the following representations: RDF, XML, OWL, DOT, GML, XGMML and SBML. Such translations are required to accommodate the different semantics of the languages in the mapping process [14], which is difficult since the semantics of OBO are not precisely defined [15].

The proposed mapping from OBO to RDF (Table 1) has undergone several refinements, not only to capture all of the OBO specification elements [16], but also to ensure a relatively natural translation, allowing users familiar with the OBOF to immediately recognise the corresponding tags. More details about the proposed format conversions can be found within the ONTO-PERL source code [17], and the entire list of RDF-ied resources that are integrated into BioGateway can be found on the project resources web page [18].

**Table 1:** OBOF to RDF mapping. Mapping used by the BioGateway pipeline to translate ontologies represented in OBOF (*e.g*. the Gene Ontology) into RDF.

OBOF2RDF	
**OBOF**	**RDF**
Term ID	<ssb:GO rdf:about="#GO_0000001">
Term name	<rdfs:label xml:lang="en">nucleus</rdfs:label>
Definition	<ssb:def>my definition</ssb:def>
Synonym	<ssb:synonym>
	<rdf:Description>
	<ssb:syn>mitochondrial inheritance</ssb:syn>
	<ssb:scope>EXACT</ssb:scope>
	<rdf:Description>
	</ssb:synonym>
Relations	<ssb:is_a rdf:resource="#GO_0048308"/>
	<ssb:part_of rdf:resource="#PO_0009070"/>
Comment	<rdfs:comment xml:lang="en">This is a comment
	</rdfs:comment>
DBXref	<ssb:DbXref>
	<rdf:Description>
	<ssb:acc>11389764</ssb:acc>
	<ssb:dbname>PMID</ssb:dbname>
	</rdf:Description>
	</ssb:DbXref>

#### BioGateway graphs

BioGateway is a system based on an RDF store that combines information from various resources [18]:

- The entire set of candidate OBO Foundry ontologies [19];
- The complete collection of annotations included in the Gene Ontology Annotation (GOA) files [20];
- A minimised version of the NCBI taxonomy [21] (including only the names, ranks, and taxonomic hierarchy);
- A subset of SWISS-PROT [22] (including only the accession numbers, synonyms, encoding genes, annotated functions and diseases); and
- The Cell Cycle Ontology (CCO) [23].

All the imported data sources, when converted to RDF graphs, share a basic URI:

http://www.semantic-systems-biology.org

This means that each resource (*e.g*. each protein from SWISS-PROT, each taxon from the NCBI taxonomy, and each OBO term) has a URI of the form:

http://www.semantic-systems-biology.org/SSB#resource

Each of the imported data sources is represented as an individual graph with a specific URI in the following form:

http://www.semantic-systems-biology.org/graph_name

Additionally, the SSB graph combines all the constituent graphs of BioGateway, containing approximately 175 million triples. Intermediate graphs for the GOA files and the OBO Foundry candidate ontologies contain approximately 160 million triples and 8 million triples, respectively (Table 2).

**Table 2:** Resource figures within BioGateway. BioGateway integrates several public resources. It holds more than 175 million triples. Intermediate graphs for the GOA files and the OBO Foundry candidate ontologies contain approximately 160 million triples and 8 million triples, respectively. These numbers are constantly increasing, depending on the new data annotations.

BioGateway figures				
**N**	**Resource name**	**Triples**	**#graphs**	**Graph name**
1	OBO foundry	7.8 m	44	OBO
2	GOA files	160 m	893	GOA
3	SWISS-PROT	4 m	1	Uniprot_sprot
4	NCBI taxonomy	2.1 m	1	ncbi
5	Biometarel	1607	1	biometarel
6	Metaonto	6865	1	metaonto

**Figure 1:**
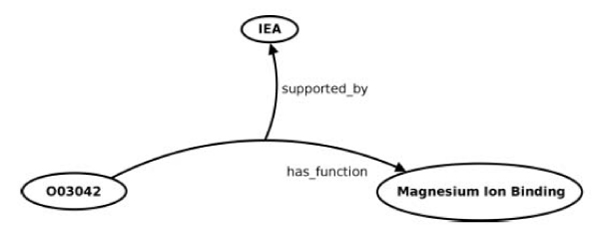
**RDF model of a GOA entry**. The protein O03042 (Ribulose bisphosphate carboxylase large chain) is annotated with the GO term GO:0000287 (Magnesium Ion Binding), a term in the Molecular Function subtree from GO. Therefore, O03042 has the molecular function of binding magnesium ion. This fact is supported by an evidence code named "Inferred from Electronic Annotation" (IEA), that is, this is an annotation that depends on computation or the automated transfer of annotations from a database (for instance, an annotation based on "hits" obtained using sequence similarity searches which were not reviewed by curators).

Many of the RDF graphs in BioGateway contain orthogonal resources that are not connected to each other, such as SWISS-PROT and the OBO Foundry ontologies. SWISS-PROT resources are, however, linked to GO resources via GOA resources. This also interlinks the three sub-ontologies of GO. To accommodate evidence codes from GOA, a reified or n-ary node is created. For example, the following excerpt from a GOA file [24] would be converted into the RDF structure shown in Figure 1:

UniProtKB O03042 O03042 GO:0000287 GOA:spkw|GO_REF:0000004 IEA

#### BioGateway scaffold: BioMetarel and Metaonto

Two ontologies were created in order to provide a scaffold for integrating all the graphs: BioMetarel and Metaonto.

BioMetarel [25] holds the predicate or relation types used to link terms. It also links the unique identifiers of the relation types with their user-friendly names. BioMetarel also contains all the meta-information, such as transitivity and reflexivity, in regard to the biomedical relation types that are used. This relation ontology consists of a generic scaffold, the Metarel ontology [26], to which the following relations are added: all the elation types of the Relation Ontology (RO) [27], and all the relation types that are used in the OBO Foundry ontologies. Unfortunately, these relation types were not consistently named throughout the candidate ontologies (*e.g*. the subsumption relation was called both: *is a* and *Is_A*, and the partonomic relation both: *part_of* and *is_part_of*). A consistent list of relation types was manually created for BioMetarel. As a rule, we chose to include a verb in every relation type name, conjugated as the third person singular in the present tense. The application of this rule predominantly involved the addition of the verb *is*. As a consequence, we can return triples in the form of a *pseudo*-grammatical sentence such as: *blood is located in vein*. This rule also prompted us to transform names such as *anatomical_relation* to: *is anatomically related to*, and *surrounding* to: *surrounds*. In fact, the meaning of several poorly named relation types became clearer by adhering to this style.

**Figure 2:**
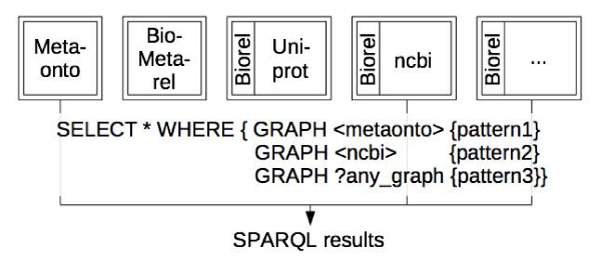
**Querying the BioGateway graph scaffold**. A copy of Biorel is added to every graph, except for BioMetarel and Metaonto. These exist as separate graphs that need to be addressed specifically within the queries.

The most straightforward use of BioMetarel is to connect the unique identifiers of the relation types with their user-friendly names. However, we observed that the inclusion of the full BioMetarel in each graph interfered with some specific queries, such as the listing of all the resources of a graph. It is more convenient for querying and exploring a graph when an RDF graph relates only to a single topic. We therefore created a lightweight subontology of BioMetarel called Biorel. This subontology contains only relation types, with no metaclasses and metarelations between relation types. This made Biorel more suitable for inclusion in every RDF graph in BioGateway. An example of a query over this scaffold is shown in Figure 2.

Having such a relation infrastructure implemented in BioGateway allowed us to build a consistent RDF scaffold for other resources, such as GOA associations together with the evidence codes. All that needed to be done to create the integrated graph was to consistently use appropriate identifiers for the predicates in the RDF triples. The integration of OBO Foundry ontologies with respect to the classes did not pose problems since these are given different identifiers in different ontologies, and they are orthogonal as a design principle.

A small ontology named Metaonto was created in the OBO format for the mapping between the names of the OBO ontologies and the prefixes they use in their unique identifiers. The mapping is helpful for users who want to explore the OBO Foundry with queries in BioGateway. Meta-information such as the names of the RDF graphs, what the graphs are about, and characteristics of the relation types are accessible as results of the so-called "ontological queries" (in contrast to "biological queries", see Section: **Queries**). In summary, the integration of data in BioGateway has been achieved based on the use of BioMetarel, the use of the same URIs for equivalent resources in the data sources (SWISS-PROT, GOA, NCBI taxonomy), and the orthogonality of OBO ontologies with respect to the classes.

### Design of BioGateway

While defining the specifications of the RDF translations for each of the integrated resources, we also developed a library of queries that were used to test performance (see Section: **Queries**). This resulted in an RDF model that is adequately suited for querying, especially in terms of performance. During this process we have paid attention to several quality constraints:

1. *Quick results:* A relatively quick query answer is always a desirable feature for any system. A query builder wants to see a quick and sound answer on a small query pattern or on a small part of the data before he launches into a heavier query. Therefore, we have systematically tested the response time with a suite of queries. This quality constraint turned out to be our biggest challenge during the development of the system. The query performance is dependent on different factors, making the subject difficult to investigate. In particular, disk access and the caching of earlier results, as well as seemingly unimportant details in the query itself can have a profound impact on the query performance, resulting in differences of one to two orders of magnitude in query time. For this reason, we removed any unnecessary chains in the RDF translations to allow short queries and to avoid inhibitive bottlenecks in the performance. The idea to provide the data in both singular and composed graphs was also inspired by performance issues. Demanding queries can now be targeted to the relevant parts of the data. Due to these optimisations, most queries described in this paper return an answer within one second, and the chance of getting an answer on a more complex query within a reasonable time are also better.
2. *Human readable output:* As RDF works with URIs, many outputs from SPARQL queries might be difficult to comprehend. We tried to avoid such outputs as much as possible by creating labels for all the terms and all the relation types that can be used to present the results to the user.
3. *Good practice:* RDF is a Semantic Web standard that implies good design practices [28] as it pertains to integration with other efforts within the framework of the Semantic Web. Orthogonality was achieved for all the terms, meaning that the proteins in SWISS-PROT received the same unique identifiers as the proteins in GOA. Otherwise, combining these graphs in a single query would not be possible.

**Figure 3:**
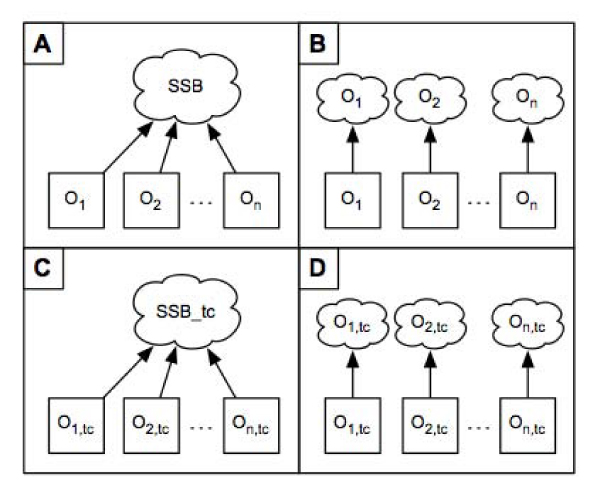
**The BioGateway graphs architecture**. Four frameworks (A-D) are implemented within BioGateway. All of them are interlinked via a common set of relations and share the same namespace: SSB. In A, all the ontologies (O_*i*_) are integrated into one single graph named SSB. In B, each ontology (O_*i*_) has its own single graph named O_*i*_. In C, all the transitive closure ontologies (O_*i*,*tc*_) belong to one single graph named SSB_tc. In D, each transitive closure ontology (O_*i*,*tc*_) has its own single graph named O_*i*,*tc*_.

A common and unique namespace called **SSB** (short for Semantic Systems Biology) was defined to gather all the resources accessible as graphs from BioGateway (Figure 3): http://www.semantic-systems-biology.org. This namespace serves as an umbrella for each piece of information stored within BioGateway; thus, a possible resource naming conflict is avoided.

#### Simulating transitive closure

Transitive closure is an important feature in biomedical knowledge representation, especially when it concerns partonomy [29]. In addition, transitive closure along the *is_a* relation type is also desirable. Transitivity cannot, however, be expressed in RDF, and therefore had to be created explicitly by adding all the necessary triples programmatically (see Section: **BioGateway architecture**). That is, if resources A, B and C are related via *part_of* (A *part_of* B *part_of* C), a third triple A *part_of* C is created. This operation was done for the candidate OBO ontologies, CCO and BioMetarel, thus allowing transitivity in queries to be exploited with little impact on the performance of BioGateway.

**Figure 4:**
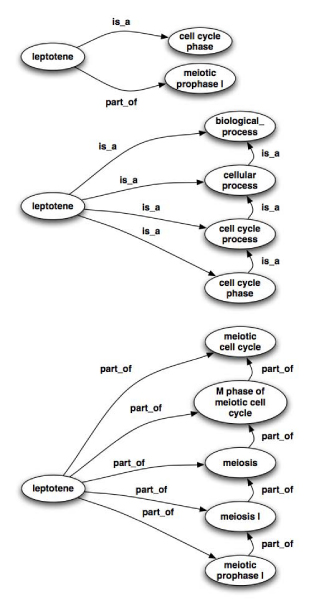
**A transitivity example**. In the case of the term *leptotene* (GO:0000237), which is originally only linked to the terms cell cycle phase (GO:0022403) via an *is_a* relation and *meiotic prophase I* (GO:0007128) via a *part_of* relation, the following implicit relations are added: *leptotene is_a biological_process* (GO:0008150), *leptotene is_a cellular process* (GO:0009987), *leptotene is_a cell cycle process* (GO:0022402), *leptotene is_a cell cycle phase* (GO:0022403), *leptotene part_of meiosis* (GO:0007126), *leptotene part_of meiosis I* (GO:0007127), *leptotene part_of meiotic prophase I* (GO:0007128), *leptotene part_of meiotic cell cycle* (GO:0051321) and *leptotene part_of M phase of meiotic cell cycle* (GO:0051327).

Transitive closure provides a scaffold for being able to reach other terms from a given term (*e.g. "* is the term *leptotene* part of the term *meiosis*?", see Figure 4). In addition, transitive closure supports queries involving compositions of relationships such as: "*if a protein P is located in L, and L is part of C and C is a D, then P is located in D"*.

#### BioGateway architecture

**Figure 5:**
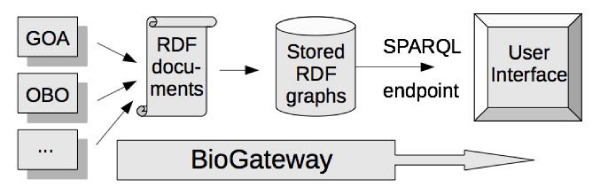
**The BioGateway pipeline**. Information resources are converted to RDF documents which are uploaded to a triple store, where they can be queried using SPARQL: First, the candidate OBO Foundry ontologies, the GOA files, the NCBI taxonomy and SWISS-PROT are integrated within the common framework defined by the in-house ontologies: BioMetarel and Metaonto. Then, for each resource, an RDF graph (and for the OBO foundry its corresponding transitive closure graph) is created and loaded into Virtuoso. This platform provides an interface (via SPARQL) to interrogate the system.

BioGateway serves as a gateway to distributed resources on the Web. A pipeline lies at the core of the system: such a pipeline automatically gathers, integrates and loads data into the RDF storage system (Figure 5). This pipeline is run every three months and builds the entire repository from scratch, ensuring integration of the latest available data. Before processing the resources to be integrated, they are automatically retrieved from their original locations so that the latest data is collected.

Initially, the complete set of candidate ontologies from the OBO foundry is processed by the pipeline. These ontologies are currently undergoing a coordinated redesign to facilitate information sharing and data analysis in the life sciences community. Thus, this set of ontologies is continuously being improved, and regular updates are needed for the BioGateway resource. All those ontologies are automatically retrieved and converted to RDF files that are then loaded into the common data repository.

The entire set of GOA files is also retrieved and integrated into BioGateway. The GOA provides connections between gene products and GO terms [30]. The integrated GOA set comprises 893 files that provide annotations for complete non-redundant proteomes covering many species. The BioGateway pipeline generates the corresponding RDF file for each of the 893 files [31]. Next, protein information from SWISS-PROT is added to BioGateway and finally, BioGateway holds the two ontologies developed in-house, Metaonto and BioMetarel that provide the scaffold for the integration and unification of the other ontologies and data (see above).

The pipeline additionally generates the transitive closure graphs (see Section: **Simulating transitive closure**) of the candidate OBO foundry ontologies, CCO and BioMetarel.

The RDF files are then uploaded into RDF graphs in Open Virtuoso [32]. Virtuoso contains an endpoint to which SPARQL queries can be submitted [33].

#### Handling of relations

The relations play a key role in the integration of different data sources. Many interesting queries (for instance, "which proteins are *located_in* the cell wall, or any *part_of* it?" or "what regulates DNA replication?") might exploit the information that is contained in the relations connecting the terms. The OBO Foundry has a policy of using a small set of shared relations for the different ontologies; the implementation of the policy is, however, far from complete. In order to create a self-contained ontology file, all relation types need to be included in the same file, with a name and a unique identifier. This has led to redundant sections for the relations of the 44 imported OBO candidate ontology files with several inconsistencies. Some relations with the same unique identifiers had different names (*e.g. part_of* and *is_part_of*). This complicates their usage, and in particular, the process of building queries over different resources that should ideally share the same relation. Moreover, the identifiers cannot serve to communicate with users, as some of them were not meant to be humanly readable, such as BSPO_0000095 (Spatial Ontology [34]) or DESCINHERM (Worm Anatomy Ontology [35]). We therefore decided not to load the redundant sections into the system and to use our own relation ontology Biorel (see Section: **BioGateway scaffold: BioMetarel and Metaonto**), which extends the relations that are used in the OBO Foundry ontologies.

We used the relations of the OBO Foundry to integrate the various data sources, including those that were not OBO-formatted. The GOA-associations essentially consist of relations between proteins in UniProt and terms in the Gene Ontology (GO). These relations were easily mapped to the OBO relation types: *has function*, *is located in*, and *participates in* for the GO molecular functions, GO cellular components and GO biological processes, respectively.

The integration of all the ontologies into the OBO Foundry allows for the detection of many engineering flaws, particularly ontologies that may be hard to find in a systematic way using other tools (see Section: **OBO foundry principles checking**). The periodic reassembly of BioGateway can therefore be exploited for quality control and the curation of the entire OBO Foundry. In addition, individual ontology engineers may find their uploaded ontology in the system, and launch dedicated queries designed for quality control.

#### OBO foundry principles checking

The OBO Foundry proposes 10 principles to which OBO bio-ontology engineers should commit [36]. That set of principles governs ontology development, and aims to ensure well-documented and redundancy-free ontologies (*i.e*. orthogonality), as well as syntactical correctness (*e.g*. OBOF specification compliance) and logical completeness (*e.g. is_a* completeness). Three of these principles can easily be checked by using BioGateway:

1. The ontologies include textual definitions for all terms.
2. The ontology uses relations that are unambiguously defined following the pattern of definitions laid down in the OBO RO.
3. The ontology must be orthogonal to other ontologies already lodged within OBO.

In particular, the orthogonality principle, that forbids the use of terms with the same name but likely with different meanings in disparate ontologies, might be difficult to check with any other tool. The following query looks for terms which have an identical name in the human disease ontology (DOID) and mouse pathology ontology (MPATH):

BASE <http://www.semantic-systems-biology.org/>

PREFIX rdfs: <http://www.w3.org/2000/01/rdf-schema#>

PREFIX ssb: <http://www.semantic-systems-biology.org/SSB#>

SELECT ?common_name ?term_id_1 ?term_id_2

WHERE {

GRAPH <human_disease> {

?term_id_1 a ssb:DOID.

?term_id_1 rdfs:label ?common_name.

}

GRAPH <mouse_pathology> {

?term_id_2 a ssb:MPATH.

?term_id_2 rdfs:label ?common_name.

}

}

The answer to the previous query highlights the term named "hyperplasia" as a repeated term. The two other listed principles can be checked in a similar way.

We have also created a query to find all the terms that have no outgoing *is_a* relationship. This can return the root terms of the ontology, but unfortunately most of the returned terms are just orphans that miss any outgoing *is_a* relationship. Many candidate OBO Foundry ontologies are not *is_a* complete. This may be due to the use of some ontology editors (*e.g*. OBO-Edit) that hide the terms in the hierarchy that have an outgoing relationship. In BioGateway, one can easily trace these terms.

### Visualisation of query results

**Figure 6:**
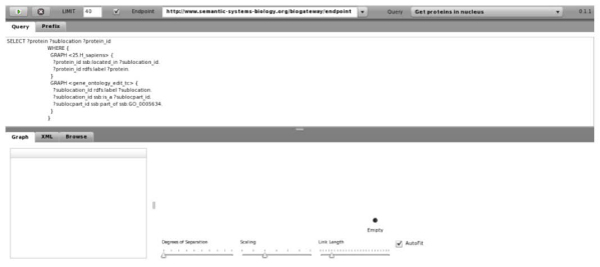
**The SPARQL viewer in BioGateway**. The queries are selected from the drop-down menu on the top right: In this case, the query "Get proteins in the nucleus" is selected. Queries can be customised, for example, by changing the parameters.

**Figure 7:**
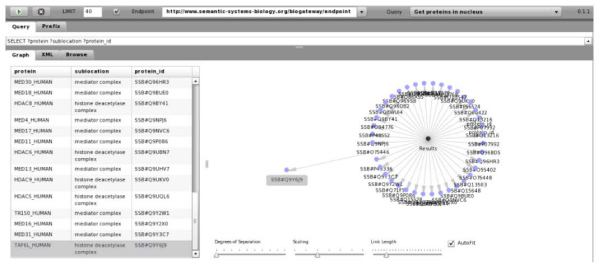
**SPARQL execution result**. The query from Figure 6 has been executed, and the results displayed. The appearance of the network can be configured.

The visualisation of triple-based resources poses a special challenge. It is necessary to develop and deploy new interfaces to manipulate, query and visualise this knowledge in an intuitive way. An SPARQL browser (still under development) enables one to query and visually explore the results obtained using BioGateway, and can be accessed from the SSB website. With this interface, users can define SPARQL queries to be launched over the resources integrated within BioGateway. The SPARQL endpoint could also be customised (by default it points to the SSB endpoint) (Figure 6). After executing a query, a network of results are displayed (Figure 7). A tabular representation of the result is also available.

### Queries

SPARQL queries can be executed against the BioGateway triple store. Many sample queries are available at the website. For example, the following SPARQL query retrieves human proteins that are located in the nucleus. The metadata about this query are presented in the first five lines at the top (lines starting with # are not interpreted by the query engine): *name*, *parameters* that can be changed and *function*; the rest constitutes the query itself (note the use of transitivity).

# NAME: get_proteins_in_nucleus

# PARAMETER: GO_0005634: the nucleus

# PARAMETER: 25.H_sapiens: the GOA graph for human

# FUNCTION: returns all the human proteins that have the

# nucleus as annotated location

BASE <> http://www.semantic-systems-biology.org/

PREFIX rdfs: <> http://www.w3.org/2000/01/rdf-schema#

PREFIX ssb: <> http://www.semantic-systems-biology.org/SSB#

SELECT ?protein ?sublocation ?protein_id

WHERE {

GRAPH <25.H_sapiens> {

?protein_id ssb:located_in ?sublocation_id.

?protein_id rdfs:label ?protein.

}

GRAPH <gene_ontology_edit_tc>{

?sublocation_id rdfs:label ?sublocation.

?sublocation_id ssb:is_a ?sublocpart_id.

?sublocpart_id ssb:part_of ssb:GO_0005634.

}

}

#### One-click query access

BioGateway provides a library of optimised, easily customisable SPARQL queries that make the resources easily accessible to both laymen users and experts, although even SPARQL experts will not easily find their way through RDF resources with which they are not acquainted. Therefore, we tried to reflect the basic query requirements in the library. This makes BioGateway accessible with a single click, and is a building block for future applications.

The library was split into a section with *biological queries* and a section with *ontological queries*. The *biological queries* are designed for use by biomedical scientists, and draw on the most relevant part of the KB. Some examples of biological queries read as follows:

1. Get the proteins with a specific function/location/process for any of the annotated organisms. For example, in Section: **Queries**, a query that returns all the human proteins that are located in the nucleus is discussed.
2. Get the information on the function, location, process and associated disease for a given protein.
3. Get the proteins that are involved in the "psoriasis" disease.

The set of *ontological queries* shows how SPARQL can be used to explore BioGateway, particularly the OBO ontologies. This set of queries is intended for users who are interested in ontology engineering. Any future applications that build on the results of SPARQL queries will certainly benefit from the availability of basic navigation-type queries such as *get neighbourhood*, *get the root of an ontology*, *get the hierarchy to the root*, *get graphs*, *etc*. These queries explore the typical network structure of RDF models. In contrast, the ontological queries show the RDF semantics that are available in BioGateway, such as subsumption, transitivity and the composition of relations. Some examples of ontological queries read as follows:

1. Query the OBO Foundry: search on names and get their unique identifiers.
2. Get all the neighbouring terms of a given term.
3. Get all the properties, such as definition, synonyms, *etc*. of a given OBO term.

Both sections of the library help to make BioGateway a workbench for creating SPARQL queries. The results of a query can often be used to copy-and-paste as a parameter in other queries. We further elaborate on this idea in the Section: **Combining regular RDF graphs with transitive closure graphs**. All the queries in the library were provided with a name, its function and a list of parameters that can be customised in a query. By properly using prefixes, an SPARQL query can be written in such a way that a parameter only needs to be replaced in one fixed place. All the queries in the library were written in this way.

#### Combining regular RDF graphs with transitive closure graphs

One of the ontological queries in the library is designed to find the closest common ancestor in the hierarchy of an ontology for two given terms:

# NAME: get_common_ancestor

# PARAMETER: GO_0002617: the first query-term

# PARAMETER: GO_0034125: the second query-term

# FUNCTION: returns the closest common ancestor-term in the

# hierarchy for two given terms

BASE <> http://www.semantic-systems-biology.org/

PREFIX rdfs: <> http://www.w3.org/2000/01/rdf-schema#

PREFIX ssb: <> http://www.semantic-systems-biology.org/SSB#

PREFIX term1_id: <SSB#GO_0002617>

PREFIX term2_id: <SSB#GO_0034125>

SELECT distinct ?common_ancestor ?common_ancestor_id

WHERE {

GRAPH <SSB_tc> {

term1_id: ssb:is_a ?common_ancestor_id.

term2_id: ssb:is_a ?common_ancestor_id.

OPTIONAL {

term1_id: ssb:is_a ?direct_child.

term2_id: ssb:is_a ?direct_child.

GRAPH <SSB> {

?direct_child ssb:is_a ?common_ancestor_id.

}

}

?common_ancestor_id rdfs:label ?common_ancestor.

}

FILTER(!bound(?direct_child))

}

For this query, we need both the regular RDF ontology and its transitive closure (**SSB_tc** that is generated by the pipeline, see Section: **BioGateway architecture**). In fact, the query might be reduced to: *find all the ancestors of both terms that do not have any descendants that are ancestral to both terms*. To find all the terms that are ancestors of both terms we need the transitive closure graph, as in that form all the ancestors are directly linked to their descendants. Two triples in the query are enough to retrieve their id:

GRAPH <SSB_tc> {

term1_id: ssb:is_a ?common_ancestor_id.

term2_id: ssb:is_a ?common_ancestor_id.

}

We find all the common ancestors with this query, while we only want the closest ones. Therefore, we check for the children of this set of ancestors. This can be best accomplished in the ontology without transitive closure:

GRAPH <SSB> {

?direct_child ssb:is_a ?common_ancestor_id.

}

Additionally, we check whether these children belong to the same set of common ancestors as defined before:

term1_id: ssb:is_a ?direct_child.

term2_id: ssb:is_a ?direct_child.

The last two checks go in an optional clause, since we only want the common ancestors for which these checks fail. In this way, we can filter the common ancestors for which this type of *?direct_child* does not exist:

FILTER(!bound(?direct_child))

#### Semantic comparative analysis

Comparative bioinformatics has yielded important new hypotheses by identifying and comparing similar features (*e.g*. genomic sequences) in different species. Comparison of these features allows the formulation of hypotheses with respect to the functions of genes and their products, as well as the localisations of gene products and the processes in which they are involved. BioGateway enables an innovative way of exploring such similarities by taking into account the stored annotations across the different organisms under consideration. The following simple query shows how this potential use can be exploited to retrieve all the proteins that have the same function (GO_0005216: *ion channel activity*), are located in the same cellular compartment (GO_0005764: *lysosome*), and participate in the same process (GO_0006811: *ion transport*) in any of the organisms of the repository:

BASE <http://www.semantic-systems-biology.org/>

PREFIX rdfs: <http://www.w3.org/2000/01/rdf-schema#>

PREFIX ssb: <http://www.semantic-systems-biology.org/SSB#>

SELECT distinct ?organism ?protein ?protein_id

WHERE {

GRAPH <SSB_tc> {

?protein_id ssb:has_function ssb:GO_0005216.

?protein_id ssb:located_in ssb:GO_0005764.

?protein_id ssb:participates_in ssb:GO_0006811.

?protein_id rdfs:label ?protein.

?protein_id ssb:has_source ?organism_id.

?organism_id rdfs:label ?organism.

}

}

ORDER BY ?organism

The query returns 11 proteins fulfilling the conditions of same function, location and process found in four organisms (*C. elegans*, *H. sapiens*, *M. musculus* and *R. norvegicus*). Not surprisingly, some of the entries are ortholog proteins (such as KCNE1_HUMAN and KCNE1_MOUSE). Some simple modifications, such as using an OPTIONAL clause over the first triple, may find that many other proteins (such as VATM_DICDI in *D. discoideum*) also share the same location and process.

## Discussion and perspectives

The life sciences community is becoming aware of the need for standards to communicate and store data and metadata [19, 37, 38]. The W3C provides standards to represent (*e.g*. RDF, OWL) and retrieve (*e.g*. SPARQL) knowledge. Although there are still limitations with respect to the representation of some types of information (*e.g*. spatio-temporal information) and it may prove difficult to model complex scenarios (such as expression data from microarray experiments), these standards have been shown to accommodate information that can be queried to gain further biological insights [39–41].

To demonstrate the utility of such standards, we built BioGateway, an RDF triple store that integrates different life sciences knowledge resources. We have shown that the use of the Semantic Web technologies makes data integration straightforward, and also allows for the enabling of flexible and fine-grained information retrieval from KBs. At the same time, we experienced some problems such as considerable upfront investment in the creation of content in RDF and performance issues while querying either very large triple stores and/or using complex queries. Other projects [42–46] have attempted similar integration. Such projects, compared to BioGateway, either used smaller data sets or offer limited query possibilities due to performance issues. They experienced similar problems. Compared to those other initiatives, BioGateway demonstrates that a sound design paves the way for reasoning and information exploitation (combination of transitive and non-transitive graphs). Thus, BioGateway provides an implicit implementation methodology that is materialised by a working system.

From our reflection upon our own experience and other similar initiatives, we identified the problems that remain to be solved in order for the Semantic Web to become a reality in Life Sciences:

### Biological identifiers

A universal resolvable mechanism for identifying biological entities is vital for a Life Sciences Semantic Web [47]. The problem of setting up a global infrastructure for biological identifiers is largely a social, not a technical one [48] as a deep agreement by the community is required. There have been different attempts in that direction. For example, the OBO Foundry insists that the ontologies have unique identifiers that are orthogonal to identifiers in other OBO Foundry ontologies. Such identifiers are, however, not resolvable, and therefore not scalable [47]. There are mechanisms that are currently proposed for resolvable identifiers such as URIs [49], LSIDs [50], OKKAM IDs [51] and MIRIAM URIs [52]. In this respect, it is appropriate to mention that the newly established group Shared Names [53] has taken up the challenge of finding a broadly acceptable solution to this problem.

### Lack of semantic content

Most biological information is either not semantically codified or it has been semantically codified with a poor axiomatisation [54]. This information should at least be semantically codified with RDF or with a rich axiomisation if using OWL. Best practices are needed to help biologists create rich semantic content in ontologies [55], so that in the future a global and distributed group of high-quality RDF/OWL encoded content will become a reality. A number of complementary approaches are being pursued to help create semantically enriched content (*e.g*. microformats [56], GRDDL [57], RDFa [58]).

### Current technology limitations

Even though ontology editors (*e.g*. Protégé [59], OBO-Edit [60]), reasoners (*e.g*. FaCT++ [61]), APIs (*e.g*. OWL API [62]) and platforms supporting the data integration (*e.g*. OpenLink Virtuoso) for the Semantic Web have advanced over the last few years, they still fall short of constituting an established and robust technology, especially in regard to their utility and reliability. On the language side, however, OWL is evolving quickly and many new features have appeared in OWL 2 [63], increasing its utility.

Academia and industry are combining efforts to bolster the development of reliable and sound Semantic Web technologies. Although research advances are more clearly appreciated at a *middleware* level (*e.g*. reasoners), that allows for the development of concrete applications such as BioGateway, scalability and visualisation (formerly relatively neglected) are becoming key aspects while developing these technologies to support the popularisation of the Semantic Web (*e.g*. the LarKC project aims to overcome reasoning limitations with vast amounts of data [64]).

The aforementioned difficulties were succinctly summarised in the title of the panel discussion during the recent SWAT4LS workshop [65]: "If the Semantic Web is so good, how come most people use OBO for ontologies and PERL for data integration?" There are various reasons for the slow pace of the adoption of Semantic Web technologies, but two of them stand out: there is a paucity of applications that demonstrate the usefulness of the Semantic Web, whereas the Semantic Web is seen as an obscure technology that is difficult to use. To illustrate this, most biologists are still unaware of the importance of semantically codifying knowledge, and perceive languages such as RDF or OWL as overly complex. They appear find the OBOF language much more suitable and intuitive than OWL or RDF. Concerning the paucity of applications, BioGateway aims at providing a demonstration of the benefits of Semantic Web technologies by facilitating a resource for querying integrated resources in order to exploit RDF.

While building BioGateway, the choice of RDF, as opposed to using OWL, resided mainly on the benefits obtained while adequately combining a set of data, a given set of queries and a query engine (OpenVirtuoso in our case). Such a combination of elements impacts many aspects, such as the speed of answers, inferencing capabilities and scalability. Some projects such as Wolfram Alpha [66], that aims to provide a "computational knowledge engine", are exploring alternative representational ways that could eventually support or complement the Semantic Web vision.

The problems observed during the development of BioGateway and other RDF stores can only be addressed at the community level. Therefore, we make a public call for the creation and development of a Semantic Systems Biology community with the following aims:

1. Encourage and facilitate the creation of semantic bio-content.
2. Develop best practices that will be commonly accepted for such content creation.
3. Collect and index such content.
4. Agree upon, and encourage a mechanism for identifying biological entities.
5. Facilitate the communication between the semantic technology developers and the life scientist: the users of such technology.

This community should have objectives beyond those of the OBO Foundry: it should build upon the best of OBO (the community, the content creation guidelines, and the content) and exploit it in a standardised platform with emerging Semantic Web qualities. As a first step towards such a community, we have built the Semantic Systems Biology wiki [67]. We venture to consider the following topics to organise and structure the life sciences Semantic Web resources, and to define a set of principles to which such a community should commit:

1. Orthogonality: avoid duplications of efforts;
2. A defined set of RDF tags (*e.g*. definition, function, has evidence, *etc*.);
3. A unique identifier per resource, plus an ID resolution (*e.g*. purl.org);
4. Comply to an accepted top-level ontology;
5. Comply to an accepted set of common relations;
6. Identify a list of prospective resource applications (*e.g*. hypothesis generation);
7. Resource peer review (community evaluation);
8. Tooling (*e.g*. visualisation);
9. Address persistence-related issues;
10. Providing explicit semantics;
11. Rich axiomatisation (and hence, rich querying).

We strongly feel that this is the appropriate moment to establish such a community to bolster and extend the current efforts (*e.g*. HCLS IG, NeuroCommons [68] that also use RDF-based triple technology, but in contrast to BioGateway in which the RDF modelling has been carefully devised to provide simple query construction, NeuroCommons uses not only RDF representations but also OWL ones) and to begin building a universal, interoperable knowledge architecture [69]. Such a structured resource will further ensure that Semantic Web technologies will become one of the most crucial means for knowledge integration in the life sciences [70, 71]. BioGateway is a demonstrative step towards such an end.

## Methods

BioGateway is built using an automated pipeline implemented in Perl (the source code is available at the SSB website [18]). This pipeline automatically integrates publicly available resources as well as in-house resources (*e.g*. Metarel).

### Sources

The following public resources are integrated under the BioGateway umbrella:

- Candidate OBO foundry ontologies (CVS repository [72], all ontologies);
- SWISS-PROT (FTP site [73]);
- NCBI taxonomy (FTP site [74]);
- GOA files (FTP site [75], all files);
- CCO (HTTP site [76]).

### Auxiliary ontologies

Metarel, Biorel and Metaonto were manually created using OBO-Edit [60]. Metarel was developed in-house to describe relations between relation types and will be described elsewhere [77]. Biorel was generated by combining RO and all the relations used in OBOF. BioMetarel was produced by adding Metarel to the relations in Biorel on their RDF translations, with SPARUL [78] – an update language for RDF graphs.

### Conversion

The conversion of GOA, SWISS-PROT and NCBI files to RDF was performed with the newly developed ONTO-PERL modules GoaToRdf, SwissProtToRdf and NcbiToRdf, respectively. The Ontology module from ONTO-PERL was used for converting ontologies from the OBO format to RDF. This conversion has been optimised, as compared to the originally published version of ONTO-PERL to minimise the number of blank nodes for the sake of query performance (see Section: **Results**). In the course of conversion, any relations present in the ontologies were omitted (to be replaced with Biorel during uploading to the triple store, see Section: **RDF endpoint**).

The conversion from SWISS-PROT and NCBI was partial content-wise. In the case of SWISS-PROT, the fields accession numbers, synonyms, encoding genes, annotated functions and diseases were retained. In the case of NCBI, the fields: taxon identifier, species, genus, family and relations were retained.

### Transitive closures

To increase the utility of the RDF representation, transitive closures were added programmatically with the use of the Ontolome module from ONTO-PERL [79]. During the transitive closure construction, each ontology term is inspected and its transitive relations (*part_of* and *is_a*) are expanded so that explicit relations to its ancestors are added (using the depth-first search algorithm [80]). This generation only considers the subsumption relation (*is_a*) and the partonomic relation (*part_of*). This was done for all OBO ontologies, CCO and BioMetarel.

### RDF endpoint

The RDF files were uploaded into the Virtuoso server using the Perl DBI module [81], the ODBC interface (DBD::ODBC [82]) and the OpenLink iODBC library [83]. All RDF files were uploaded as individual graphs. In the course of uploading, four additional integrated graphs were created: the **SSB** graph including all individual graphs, the **SSB_tc** graph containing all the transitive closures, the **OBO** graph comprising all the OBO candidate ontologies and the **GOA** graph combining all the GOA graphs. A copy of the **Biorel** graph was added during the upload to each of the graphs (except Biorel and BioMetarel).

### Web interface

A web interface [84] for querying BioGateway with SPARQL was developed using the Joomla content management system [85] and JavaScript. This interface provides a set of pre-cooked queries showing sample query possibilities on the system, and an edit-box (form) that points to the SPARQL endpoint through simple HTML technology.

### Graph visualisation

The SPARQL Browser [86], a web application for displaying query results, was developed with Flex technologies [87]. The source code is available at http://www.netthreads.co.uk.
